# Peaches Preceded Humans: Fossil Evidence from SW China

**DOI:** 10.1038/srep16794

**Published:** 2015-11-26

**Authors:** Tao Su, Peter Wilf, Yongjiang Huang, Shitao Zhang, Zhekun Zhou

**Affiliations:** 1 Key Laboratory of Tropical Forest Ecology, Xishuangbanna Tropical Botanical Garden, Chinese Academy of Sciences, Mengla 666303, China; 2 State Key Laboratory of Paleobiology and Stratigraphy, Nanjing Institute of Geology and Paleontology, Chinese Academy of Sciences, Nanjing 210008, China; 3 Department of Geosciences, Pennsylvania State University, University Park, Pennsylvania 16802, USA; 4 Key Laboratory for Plant Diversity and Biogeography of East Asia, Kunming Institute of Botany, Chinese Academy of Sciences, Kunming 650204, China; 5 Faculty of Land Resource Engineering, Kunming University of Science and Technology, Kunming 650093, China

## Abstract

Peach (*Prunus persica*, Rosaceae) is an extremely popular tree fruit worldwide, with an annual production near 20 million tons. Peach is widely thought to have origins in China, but its evolutionary history is largely unknown. The oldest evidence for the peach has been Chinese archaeological records dating to 8000–7000 BP. Here, we report eight fossil peach endocarps from late Pliocene strata of Kunming City, Yunnan, southwestern China. The fossils are identical to modern peach endocarps, including size comparable to smaller modern varieties, a single seed, a deep dorsal groove, and presence of deep pits and furrows. These fossils show that China has been a critical region for peach evolution since long before human presence, much less agriculture. Peaches evolved their modern morphology under natural selection, presumably involving large, frugivorous mammals such as primates. Much later, peach size and variety increased through domestication and breeding.

Peach (*Prunus persica*, Rosaceae) is a universally known tree fruit with an annual production near 20 million tons^1^; it is also a genetic model organism^2^. China has a long history of peach cultivation known from both historical and archaeological evidence^3^,^4^. The word peach (“
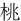
”) has long appeared in Chinese literature, e.g., the books *Shi-Jing* (1,100–600 BC) and *Shi-Ji* (1st century BC)^3^. Despite the significant fossil record of Rosaceae and the genus *Prunus*^5^,^6^,^7^,^8^,^9^,^10^,^11^,^12^,^13^,^14^,^15^, the origins of the peach and its unique features remain unknown. No wild population has been confirmed^16^, and the long history of trade and complex genomics of peach cultivars present considerable obstacles^17^. Recently, we found eight fossil peach endocarps, in the late Pliocene Ciying Formation in Kunming, Yunnan, southwestern China (Fig. 1), whose morphological characters are identical to modern peaches. This discovery of the oldest fossil peaches provides important evidence for the origins and evolution of the modern fruit.

**Order**—Rosales Bercht. & J. Presl

**Family**—Rosaceae Juss.

**Genus**—*Prunus* L.

**Subgenus**—*Amygdalus* L.

**Species**—*Prunus kunmingensis* T. Su, P. Wilf et Z.K. Zhou sp. nov.

**Holotype**—KUN PC2015001 (Fig. 2a) (designated here).

**Paratypes**—KUN PC2015002 (Fig. 2b), KUN PC2015003 (Fig. 2c), KUN PC2015004 (Fig. 2d), KUN PC2015005 (Fig. 2e), KUN PC2015006, KUN PC2015007, KUN PC2015008.

**Locality**—The late Pliocene Ciying Formation, North Terminal Bus Station, Kunming, central Yunnan Province, southwestern China (Fig. 1).

**Repository**—The Herbarium of Kunming Institute of Botany, Chinese Academy of Sciences (KUN).

**Etymology**—The specific epithet "*kunmingensis*" refers to the discovery location in Kunming.

**Description**—Stony endocarps (Fig. 2) elliptical, flattened in lateral view (presumably compressed), base obtuse, apex apiculate, length 2.6–3.0 cm, width 1.8–2.3 cm, length:width ratio 1.3–1.6:1, thickness 0.8–1.2 cm. Endocarp exterior surface with both furrows and pits. Single deep groove of vascular bundle canal on dorsal side, extending from base to apex. Ridge on ventral side. Transverse furrows (Fig. 2a) one or two, following edges of both dorsal and ventral sides. Longitudinal furrows (Fig. 3a) seven to ten, radiating apically from the base over less than half the endocarp length. Deep pits (Fig. 2) mainly situated near the apex. Endocarp interior surface (Fig. 3c) smooth, with linear striations; internal sclerids (Fig. 3e) apparently diagenetically altered. Seed (Fig. 2f) single, flattened, elliptical, base round, apex acute, length ~1.9 cm, width ~1.0 cm, replaced by iron compounds.

## Discussion

Several characters of the fossils, including the large, single-seeded endocarp, elliptic shape, and the deep vascular bundle canal along the edge of the dorsal side, unambiguously assign them to the genus *Prunus*^8^. Although molecular phylogenies are revising infrageneric relationships^18^, Rehder’s^19^ widely-used classification of *Prunus* recognizes five subgenera: *Amygdalus*, *Cerasus*, *Laurocerasus*, *Padus*, and *Prunus.* Of these, subgenus *Amygdalus* is consistent with the fossils in having the largest endocarp size (mean lengths and widths usually more than 1.5 cm^16^,^20^) and because it is the only subgenus with species that have both furrows and pits on the endocarps (Figs 2 and 3a).

Subgenus *Amygdalus* has two sections, *Amygdalus* and *Persica*^21^. Endocarp shape and size are similar in both sections, but deep furrows are usually absent in section *Amygdalus*^21^. Many additional features of the fossils show their close affinity to the living peach, *Prunus persica*, as seen in our full character matrix for 36 modern *Prunus* species, plus the fossils, that shows identical scores for the fossils and modern peaches (Fig. 4; Supplementary Table 1). The most distinctive features of peaches that are seen in the fossil endocarps, in combination, are the large size, apiculate apex, presence of both pits and furrows on the exterior surface, and typical linear striations on the interior endocarp surface (Fig. 3). In sum, the well-preserved fossil endocarps show no differences from the living peach (Supplementary Figure 1) and could be assigned to the extant species. However, other organs of the ancient plant are not yet known, and we instead propose the new species name, *Prunus kunmingensis*, to provide an unambiguous epithet for the fossils in the absence of a whole-plant reconstruction.

The well-preserved specimens reported here comprise the earliest record of peach, from the late Pliocene (i.e., by ca. 2.6 million years ago), as well as the only occurrence that predates archaeological evidence. The oldest reliable evidence for the genus *Prunus* comes from the Eocene of the Northern Hemisphere as endocarps, leaves, and wood, as recently reviewed by DeVore and Pigg^22^. Nevertheless, there are no reliable fossils that show close morphological similarities to peach^8^,^15^ except for subfossils that are mostly from Chinese archaeological sites^3^,^4^, because the typical characters of endocarps in peach, i.e., the presence of deep pits and furrows, as well as the apiculate apex, are absent in all these fossils.

The associated flora from the fossil-bearing layer, as well as global and regional paleoclimate reconstructions, all indicate that the ancient peach trees lived in a warmer, wetter regional climate than today^23^,^24^,^25^. The associated flora includes abundant fruits of the ring-cupped oak (*Quercus* subgenus *Cyclobalanopsis*, Fagaceae; Supplementary Figure 2), whose extant species are evergreen trees that principally inhabit tropical and subtropical Asian forests. Both ring-cupped oak and *Prunus davidiana*, a species with close affinity to peach, are found naturally today in subtropical forests of central Yunnan Province^26^.

*Prunus kunmingensis* demonstrates the early presence of peach in southwestern China and dramatically increases the region’s established significance for the evolutionary origins and cultivation history of the fruit. Southwestern China holds high species diversity in rosaceous genera with agricultural significance such as *Malus* (apple), *Prunus* (almond, apricot, cherry, peach and plum), and *Pyrus* (pear) (Supplementary Figure 3). In *Prunus* section *Persica*, all species except for *Prunus mongolica* are native to the region^16^, and a natural population of *Prunus mira* (section *Persica*), with some individual trees more than 1000 years old, exists in Linzhi County, eastern Tibet^27^. That region is also especially rich in local peach cultivars^27^,^28^.

The endocarps of modern peach cultivars show much more morphological variation and generally larger sizes than both *Prunus kunmingensis* and archaeological subfossils (Supplementary Figure 4), presumably reflecting the subsequent selection of varieties under cultivation. However, the size of *Prunus kunmingensis* is within the lower range of modern peach cultivars (Fig. 5, Supplementary Table 2), as are peach endocarps from some archaeological sites^3^,^4^. In modern peaches, endocarp size positively correlates with fruit size (Fig. 5); if this correlation existed in the past, the fossil endocarps indicate mean fruit diameters of ~5.2 cm (Fig. 5).

Presumably, the fleshy ancient peaches would have been a desirable food source for large-bodied frugivores such as primates. Of special interest, the fossils show that peaches were already present in SW China before the Pleistocene arrivals of *Homo erectus* and *Homo sapiens*^29^. The universally known seed-dispersal mutualism between hominids and peaches, in all likelihood, is very ancient.

## Methods

### Geological setting

**Figure 1:**
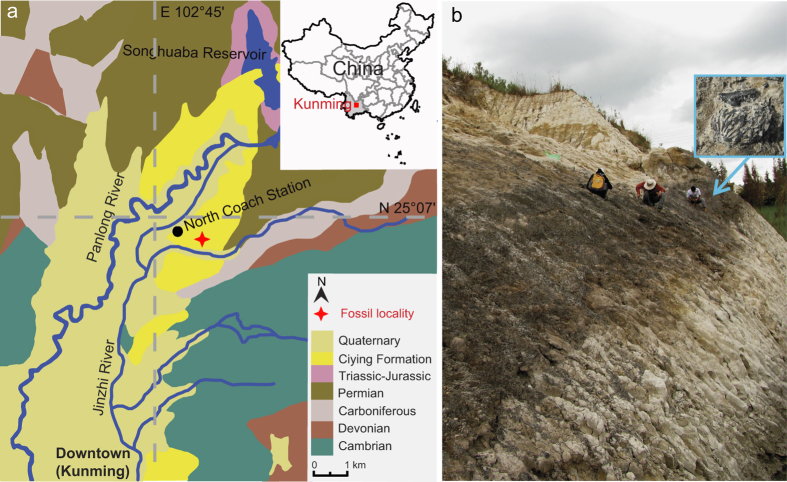
Fossil locality in Kunming, Yunnan Province, southwestern China. (**a**) Geologic map, modified from Bureau of Geology and Mineral Resources of Yunnan Province, 1990^30^ with the software Adobe Illustrator CS4. (**b**) Stratigraphic section, arrow showing the fossil-bearing layer; inset shows fossil peach endocarp *in situ*.

**Figure 2:**
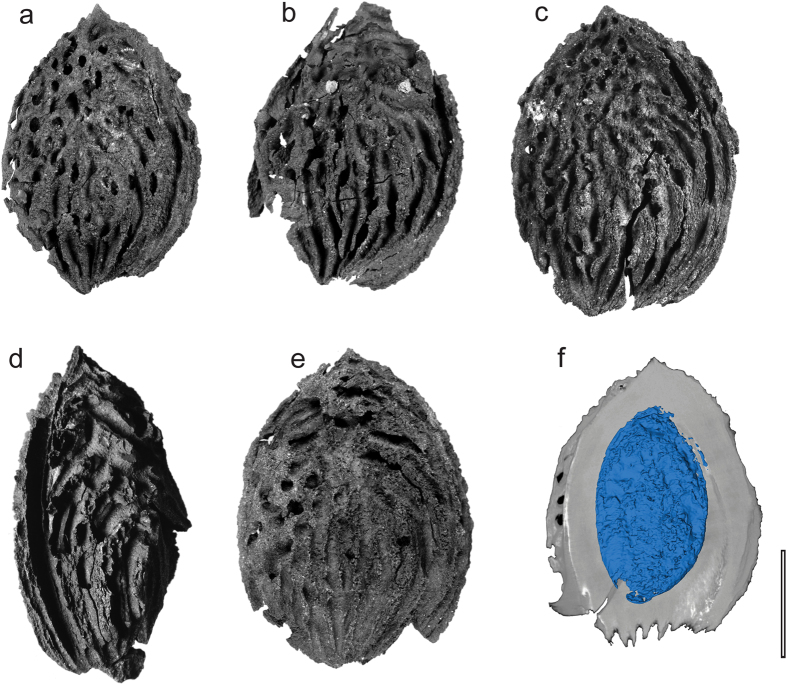
*Prunus kunmingensis*. (**a**–**e**) KUN PC2015001-KUN PC2015005. (**f**) CT scan showing longitudinal section and seed (KUN PC2015001). Scale bar = 1 cm. See the three dimensional reconstruction in Animation S1.

During August, 2010, eight fossil fruit endocarps were collected near North Terminal Bus Station of Kunming, Yunnan Province, southwestern China (25° 06'19.77"N, 102°45'52.45"E, 1974 m a.s.l.; Fig. 1a) by Paleoecology Group members of Xishuangbanna Tropical Botanical Garden. The fossiliferous strata, recently exposed by new road construction (Fig. 1b), are assigned to the Ciying Formation^30^. The stratigraphy of the Ciying Formation has been extensively described^31^. The geological age of the formation is considered to be late Pliocene^32^ based on a combination of lithostratigraphic correlations^30^, paleomagnetic data^33^, and regional palynology^34^. The fossils studied here came from organic-rich silty mudstones in the upper layer of the formation.

### Morphological observation

Fossils were soaked in distilled water and cleaned with an ultrasonic cleaner (UC KO-50M) at a frequency of 40 kHz for 10 minutes to remove sand grains on the surface. Macrophotography was done with a Nikon D700 on a Kaiser 5510 stand, with the fossils placed on glass to eliminate shadows and improve contrast. Measurements were taken with a digital vernier caliper (Mitutoyo 500–351). The surface features of the fossils were observed under a stereo microscope (Zeiss REO Discovery V20). Detailed morphology was obtained under a scanning electron microscope (Zeiss EVO LS10) in the Central Laboratory, Xishuangbanna Tropical Botanical Garden, Chinese Academy of Sciences. To observe three-dimensional internal structures, the fossils were scanned with a HD-600 CT scanner in the Center for Quantitative X-Ray Imaging at Pennsylvania State University.

Morphology of modern *Prunus* endocarps was examined at the U.S. National Seed Herbarium (located at the U.S. National Arboretum, Washington, DC) and the Herbarium of Kunming Institute of Botany (KUN). For each species, one to five fruit endocarps (average of three) were observed, depending on the number of specimens available. Twelve morphological characters were scored for the endocarps of the fossils and for 36 living *Prunus* species (Supplementary Table 1). Nonmetric Multidimentional Scaling with euclidean distance measure, using the software PAST (Version 1.75 b, Oslo, Norway) was applied to analyze morphological similarity among species (Fig. 4).

### Exclusion of modern sample contamination

**Figure 3:**
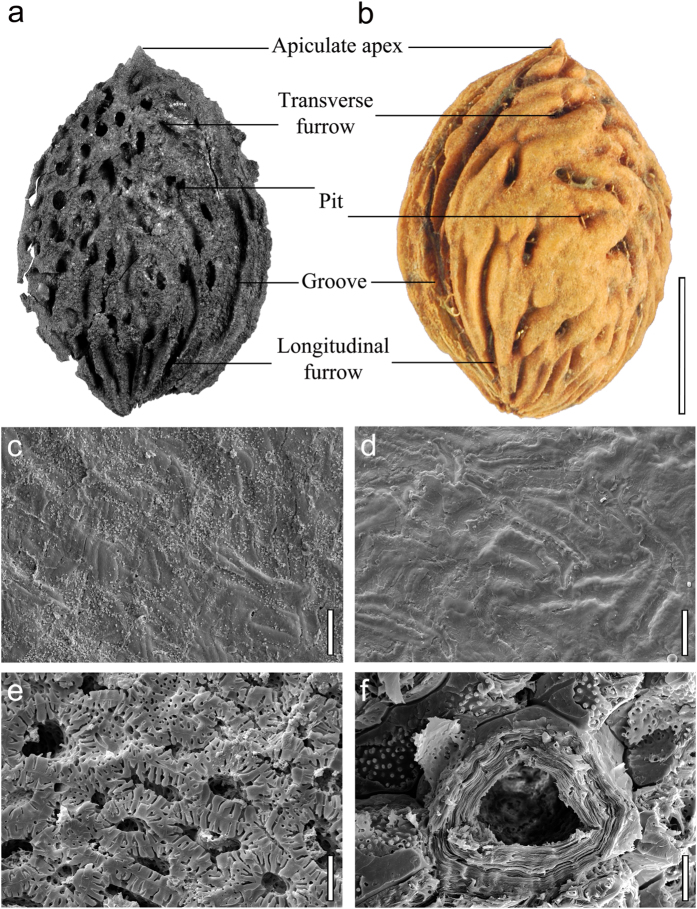
Morphological comparison of endocarps between *Prunus kunmingensis* (**a**,**c**,**e**) and modern peach (**b**,**d**,**f**). (**a**,**b**) Gross morphology. (**c**,**d**) Endocarp interior surface with linear striations. (**e**,**f**) Diagenetically altered fossil sclereids (**e**) and modern sclereids (**f**) along a transverse section of the endocarp. Scale bars: a–b = 1 cm; c–d = 30 μm; e–f = 15 μm.

**Figure 4:**
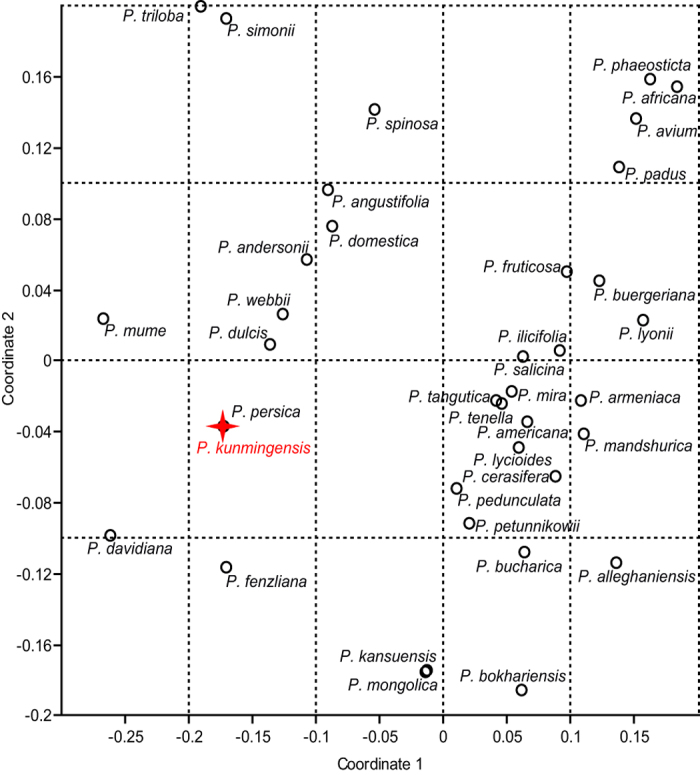
Nonmetric Multidimentional Scaling analysis of 36 *Prunus* species based on 12 morphological characters of endocarps. *Prunus kunmingensis* and modern peach share the same character scores. Data listed in Supplementary Table 1.

**Figure 5:**
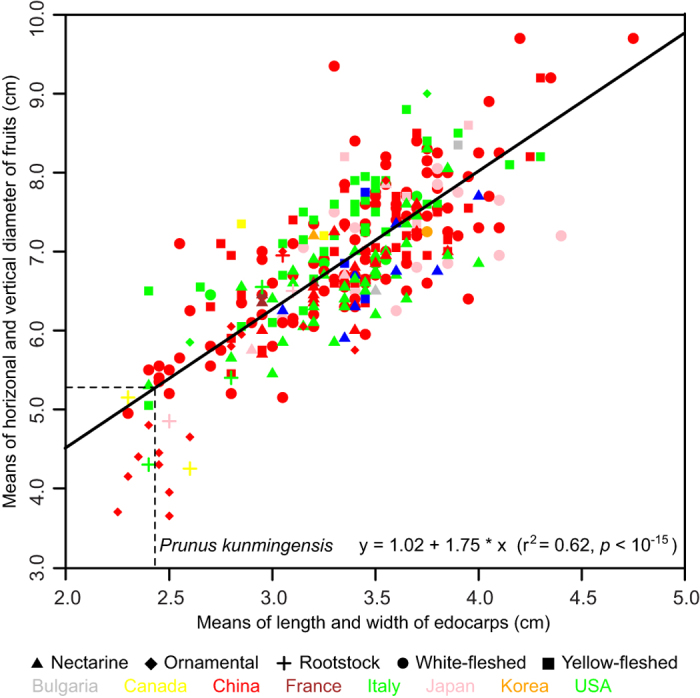
Size correlation between endocarps and fruits in modern peach cultivars and estimated fruit size in the fossil *Prunus kunmingensis*. Data from modern cultivars are measured from photographs in Wang *et al.* 2012^28^ and listed in Supplementary Table 2. Lines indicate the fossil endocarps of Prunus kunmingensis, whose inferred fruit diameter, based on the correlation shown here (solid black line), is ~5.2 cm.

Fossils were extracted from freshly exposed, well-bedded strata on a steep slope, in a layer containing many other species of fossil plants. However, the fossils look so strikingly modern that the possibility of modern contamination needed to be rigorously excluded. We measured the elemental composition of a seed in one of the endocarps, using Energy Dispersive analysis (EDS) with a scanning electron microscope (FEI Nova NanoSEM 630) in the Materials Characterization Laboratory at Pennsylvania State University. The seed is mostly replaced by iron oxides (Supplementary Table 3). In addition, we analyzed the ^14^C age of one endocarp at Beta Analytic (Miami, USA), using the AMS-Standard delivery method (Supplementary Table 3). Results indicate that the age of the fossils is beyond the range of radiocarbon dating (>43,500 years, Supplementary Table 3). The endocarps are flattened, presumably from compression, and the sclereid morphology is diagenetically altered (Fig. 3e), which further confirmed that the fossils are ancient and do not represent recent human activities at the site. All these lines of evidence show that the fossils were preserved within the Pliocene strata and do not represent more recent additions.

## Additional Information

**How to cite this article**: Su, T. *et al.* Peaches Preceded Humans: Fossil Evidence from SW China. *Sci. Rep.* **5**, 16794; doi: 10.1038/srep16794 (2015).

## Supplementary Material

Supplementary Information

Supplementary Animation
