# Supplementary material for: Peaches Preceded Humans: Fossil Evidence from SW China
Source: Sci Rep. 2015 Nov 26;5:16794. doi: 10.1038/srep16794 (PMC4660870; doi:10.1038/srep16794)
Supplement: Supplementary Information [file srep16794-s1.pdf]

Peaches Preceded Humans: Fossil Evidence from SW China

Tao Su[1, 2], Peter Wilf[3], Yongjiang Huang[2, 4], Shitao Zhang[5], Zhekun Zhou[1, 4]

[1]Key Laboratory of Tropical Forest Ecology, Xishuangbanna Tropical Botanical Garden, Chinese Academy of Sciences, Mengla 666303, China. [2]State Key Laboratory of Paleobiology and Stratigraphy, Nanjing Institute of Geology and Paleontology, Chinese Academy of Sciences, Nanjing 210008, China. [3]Department of Geosciences, Pennsylvania State University, University Park, Pennsylvania 16802, USA. [4]Key Laboratory for Plant Diversity and Biogeography of East Asia, Kunming Institute of Botany, Chinese Academy of Sciences, Kunming 650204, China. [5]Faculty of Land Resource Engineering, Kunming University of Science and Technology, Kunming 650093, China.

Correspondence and requests for materials should be addressed to Z.K.Z. (email: zhouzk@xtbg.ac.cn).

Supplementary Online Materials

Supplementary Table 1 │ Morphological character matrix for *Prunus kunmingensis* and 36 living *Prunus* species.

Supplementary Table 2 │ Endocarp size in *Prunus kunmingensis* (this study) and archaeological fossils[4], and endocarps and fruit size in 302 modern peach cultivars[29].

Supplementary Table 3 │ Results of ^{14}C dating and elemental analysis for *Prunus kunmingensis*. Radiocarbon dating was done in Beta Analytic Inc. (July, 2013), and elemental analysis of one seed of *P. kunmingensis* was carried out in the Materials Characterization Laboratory at Pennsylvania State University (November, 2014).

Supplementary Animation 1 │ Three dimensional reconstruction of a fossil endocarp (KUN PC2015001) from CT data, showing external and internal structures.

Supplementary Figure 1 │ Phylogeny of *Prunus* section *Persica* (redrawn from Yazbek and Oh, 2013[22]) and a summary comparison of endocarp morphology, including the *P. kunmingensis* fossils.

Supplementary Figure 2 │ Ring-cupped oak from the fossil-peach layer. a and b. Fruits with cupule. c. Spiral bracts on the cupule. d-f. Fruits. Scale bars: c = 1 mm; others = 1 cm.

Supplementary Figure 3 │ The proportions of species native to southwestern China in selected economically important genera of Rosaceae. Numbers indicate

how many species are native to southwestern China and all-China, respectively. “Southwestern China” includes Chongqing, Guizhou, Sichuan, southeastern Tibet, and Yunnan, together comprising about 13% of the Chinese land area.

Supplementary Figure 4 │ Examples of morphological diversity among endocarps in modern peach cultivars. Specimens were examined at the U.S. National Seed Herbarium. Specimen numbers: a. SPI.65856; b. SPI.41498; c. SPI.41732; d. FPI.102706; e. SPI.131406; f. SPI.43290; g. SPI.41733; h. SPI.36703; i. SPI.41274; j. SPI.43289. Scale bar = 1 cm.

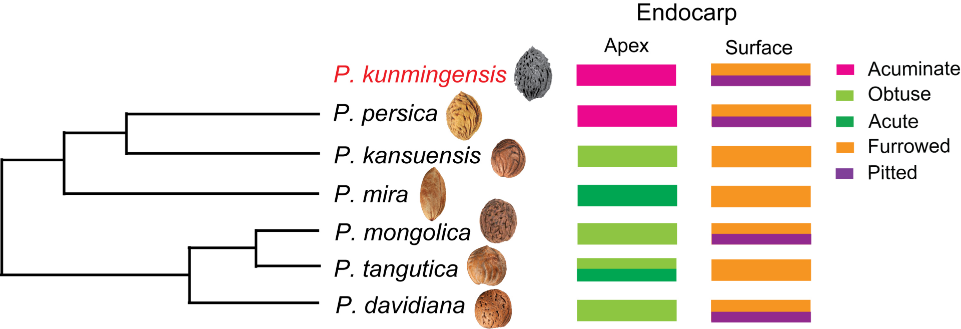
Endocarp
Apex
Surface
P. kunmingensis
P. persica
P. kansuensis
P. mira
P. mongolica
P. tangutica
P. davidiana
Acuminate
Obtuse
Acute
Furrowed
Pitted

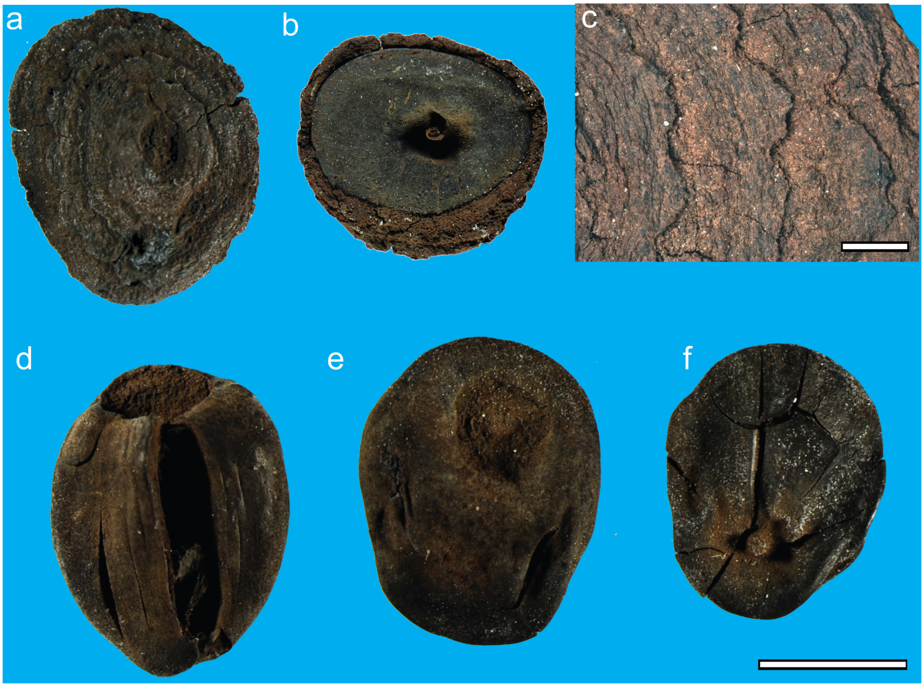
a
b
c
d
e
f

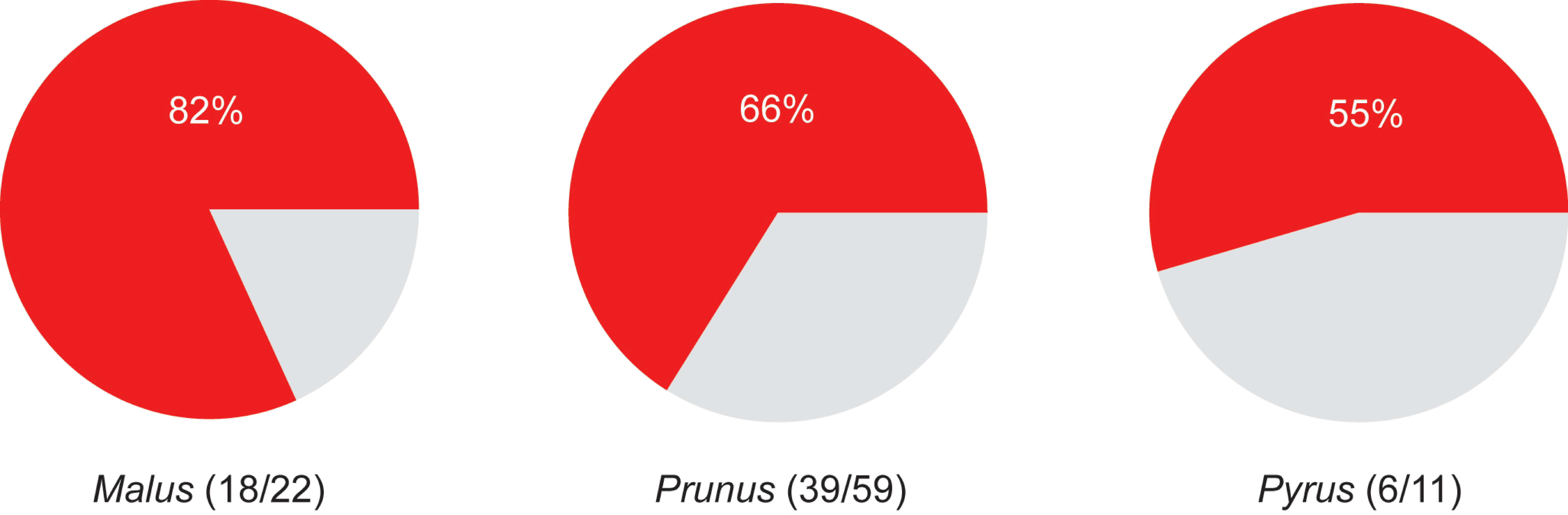
82%
66%
55%
Malus (18/22)
Prunus (39/59)
Pyrus (6/11)

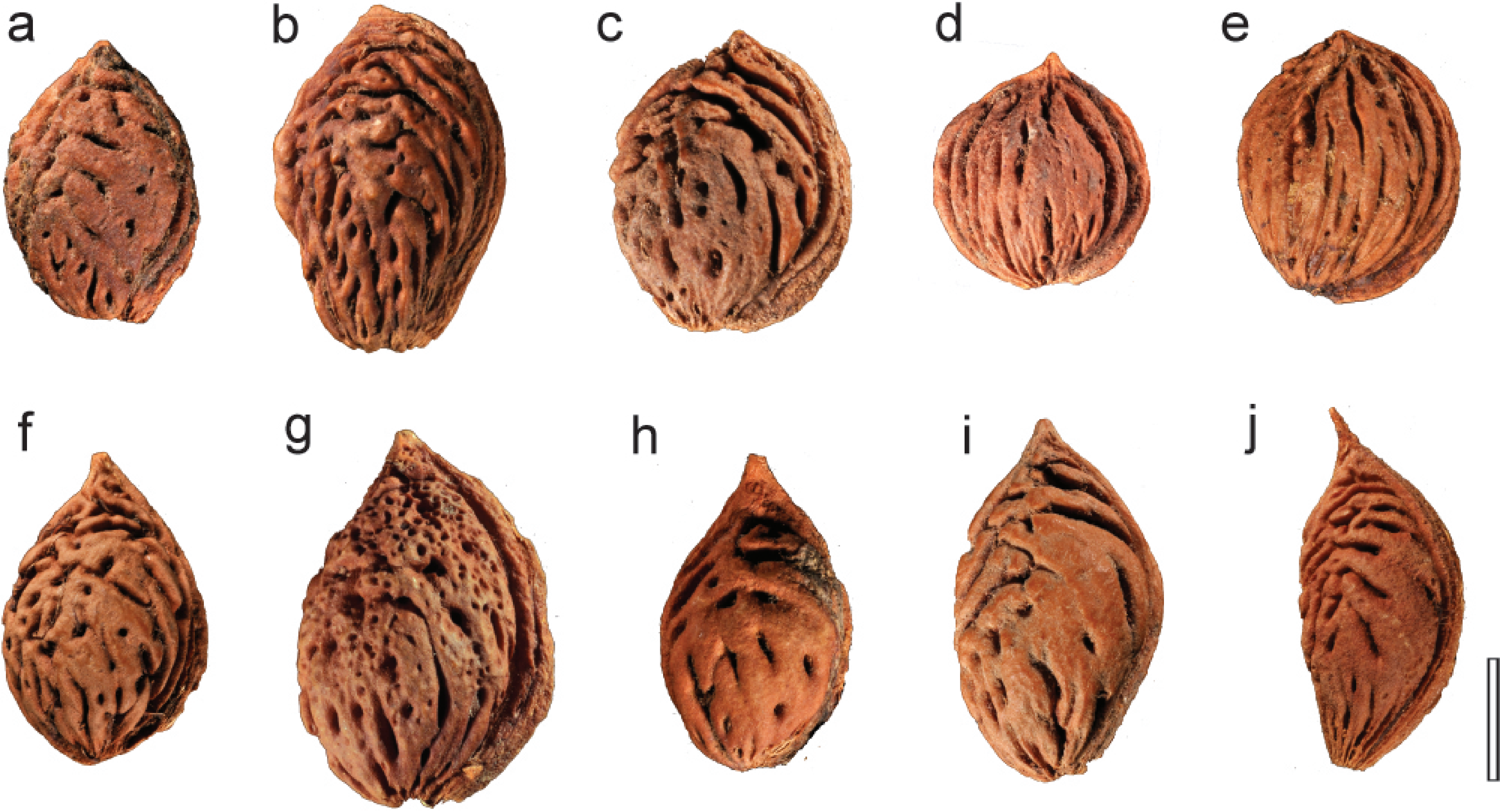
a
b
c
d
e
f
g
h
i
j

Species	Voucher number	Shape of endocarp	Pit (frequency)	Pit (shape)	Basal furrow	Transverse furrow	Apex of endocarp	Base of endocarp	Length:Width	Length	Width	Groove of vascular bundle canal	Position of vascular bundle canal
		Elliptic=0; Round=1	Absent=0; Few=1; Many=2	Absent=0; Shallow=1; Deep=2	Absent=0; Present=1	Absent=0; Present=1	Round=0; Acute =1; Acuminate =2; Obtuse=3	Round=0; Acute=1; Obtuse=2	<1:1 = 0; ⩾1:1 =1	<1cm = 0; ⩾ 1cm<2cm =1; ⩾2cm =2	<1cm = 0; ⩾ 1cm<2cm =1; ⩾2cm =2	Shallow=0; Deep=1	Not near the margin =0; Near the margin=1
Prunus kunmingensis	KUN PC2015001-8	0	1	2	1	1	2	2	1	2	1,2	1	1
P. persica	SPI. 41419; SPI. 41274; SPI. 41398; SPI. 41497; SPI. 41498	0	1	2	1	1	2	2	1	2	1,2	1	1
P. africana	P.I. 116694	1	0	0	0	0	0	0	0	0	0	0	1
P. alleghaniensis	USNHSC 4469	0	0	0	0	0	2,3	1	1	0,1	0	0	0,1
P. americana	USDASH 46231	0	0	0	0	0	1,3	1	1	1	1	1	1
P. andersonii	IP. 6452; SPI. 21657; SPI. 26297; USNHSC 35909	0	2	1	0	0	2	1	1	1	0,1	1	1
P. angustifolia	Econ. Coll.	0	2	1	1	0	1	2	1	1	0	1	1
P. armeniaca	SPI. 62632	1	0	0	1	0	1	2	0,1	1	1	1	0
P. avium	P.I. 126527; P.I. 128826; P.I. 125625; P.I. 124926; SPI. 65127	0,1	0	0	0	0	0	0	1	0	0	0	1
P. bokhariensis	P.I. 137729	1	0	0	0	0	3	2	1	1	0,1	1	0
P. bucharica	PI. 113734; SPI. 30975	0	0	0	0	0	2	2	1	2	1	0	0
P. buergeriana	FPI. 81550	1	0	0	0	0	1,2	2	1	0	0	0	1
P. cerasifera	P.I. 125629; P.I. 324007; P.I. 324165; P.I. 324166; P.I. 324008	0	0	0	0	0	1,3	2	1	2	1	0	0
P. davidiana	SPI. 22009; SPI. 47949; SPI. 49409; SPI. 52662; SPI. 52849	1	2	2	1	1	3	2	0,1	1,2	1	0	1
P. domestica	P.I. 324011; P.I. 324012	0	2	1	0	0	1,3	2	1	1,2	0,1	1	1
P. dulcis	SPI. 28802; SPI. 29214; SPI. 40213; SPI. 50117; SPI. 56177	0	1	2	1	0	1,2	2	1	1,2	1,2	0	1
P. fenzliana	SPI. 27336; SPI. 43302	0	1	2	0	1	3	2	1	1	0,1	0	1
P. fruticosa	P.I. 324013	0	0	0	1	0	1	2	1	0	0	0	1
P. ilicifolia	2019	1	0	0	0	0	1	2	1	1	1	0	1
P. kansuensis	FPI. 100509; SPI. 39428; SPI. 40864; SPI. 68976	1	0	0	1	1	3	2	1	1	1	1	1
P. lycioides	FPI. 28943; FPI. 28944	0	0	0	0	0	2	2	1	1,2	0,1	0	1
P. lyonii	?	1	0	0	0	0	0,1	1	1	1,2	1	0	1
P. mandshurica	P.I. 324014; P.I. 324016; P.I. 324017;	0	0	0	0	0	1	1	1	1,2	1	0	0
P. mira	SPI. 34601	0	0	0	1	0	1	1	1	1	1	1	1
P. mongolica	?	1	0	0	1	1	3	2	1	1	1	1	1
P. mume	SPI. 28685	0	2	2	1	0	2	2	1	2	2	1	0
P. padus	P.I. 317265	0	0	0	0	1	0	2	1	0	0	0	1
P. pedunculata	SPI. 65686	0	0	0	1	1	2	2	1	1	0	1	1
P. petunnikowii	SPI. 73602	0	0	0	1	1	2	1	1	1,2	1	0	1
P. phaeosticta	P.I. 325016	1	0	0	0	1	0	0	1	0	0	0	1
P. salicina	P.I. 324020; SPI. 37006	0	0	0	0	0	1	2	1	1	1	1	1
P. simonii	SPI. 39438	1	2	2	1	0	0	2	1	1	1	1	0
P. spinosa	P.I. 324021; PI. 324021	0	2	1	0	0	1	2	1	0,1	0	1	1
P. tangutica	SPI. 40010	0	0	0	1	1	1,3	2	0,1	1	1	0	1
P. tenella	SPI. 32760	0	0	0	1	0	1	2	1	2	1	0	1
P. triloba	PI. 115091	1	2	2	1	1	0	0	1	1	1	1	1
P. webbii	PI. 108768	0	1	2	1	1	1	2	1	1	1	0	1

		Endocarp (cm)			Fruit (cm)			Weight (g)
Cultivar or fossils	Site	Width	Length	Average	Width	Length	Average	
Prunus kunmingensis	Kunming, Yunnan, China	2.0	2.8	2.4				
Archaeological record	Kuahuqiao, Zhejiang, China	2.0	1.6	1.8				
Archaeological record	Tianluoshan, Zhejiang, China	1.9	1.5	1.7				
Archaeological record	Maoshan, Zhejiang, China	2.2	1.7	2.0				
Archaeological record	Bianjiashan, Zhejiang, China	2.0	1.6	1.8				
Archaeological record	Qianshanyang, Zhejiang, China	2.6	1.9	2.3				
Archaeological record	Ikiriki, Japan	2.2	1.8	2.0				
21 Shi Ji Tao	Hebei, China	2.8	4.3	3.6	8.3	8.1	8.2	180
C4R2T23	USA	3.1	4.3	3.7	8.0	7.4	7.7	208
D2R32T158	USA	2.9	4.4	3.7	7.4	8.1	7.8	91
Abehakuto	Japan	2.6	4.0	3.3	7.5	7.5	7.5	184
Anlong Bai Tao	Guizhou, China	3.1	4.0	3.6	7.0	6.3	6.7	108
Anlong Shui Mi	Anhui, China	3.2	5.5	4.4	8.9	9.5	9.2	213
Hakuho	Japan	3.1	4.1	3.6	6.7	5.8	6.3	128
Bai He Tao	Yungui Plateau, China	2.9	4.0	3.5	7.8	7.4	7.6	134
Bai Hua	Jiangsu, China	3.0	4.6	3.8	7.7	7.9	7.8	155
Bai Li Hu	Yunnan, China	3.1	4.8	4.0	6.5	6.3	6.4	123
Bai Nian He	Yunnan, China	3.3	4.9	4.1	7.3	7.3	7.3	152
Bai Sha	Shaanxi, China	2.8	4.1	3.5	6.6	7.2	6.9	130
Bai Xiang Lu	Jiangsu, China	3.3	4.6	4.0	7.7	8.2	8.0	120
Snow Princess	USA	2.0	3.4	2.7	6.3	6.6	6.5	150
Bao Lu	Zhejiang, China	2.9	4.7	3.8	7.7	8.3	8.0	160
Beijing 11-12	Beijing, China	2.5	2.9	2.7	5.7	5.4	5.6	50
Beijing 21-2	Beijing, China	2.5	3.9	3.2	6.4	6.0	6.2	49
Beijing 76-9-1	Beijing, China	2.7	4.0	3.4	6.8	6.6	6.7	112
Bei Nong 2	Beijing, China	3.2	4.1	3.7	7.0	6.7	6.9	138
Bei Nong Zao Shu	Beijing, China	2.3	3.3	2.8	5.4	5.0	5.2	50
Nunome Wase	Japan	3.4	4.3	3.9	6.5	7.2	6.9	130
Kurakato Wase	Japan	3.1	4.5	3.8	7.6	7.4	7.5	150
Changling Zao Yu Lu	Zhejiang, China	3.0	4.5	3.8	7.8	8.2	8.0	137
Nagasawa Hakuho	Japan	2.7	4.1	3.4	6.7	6.3	6.5	135
Chi Yuan Mi	Jiangsu, China	3.0	5.1	4.1	7.6	7.8	7.7	180
Hatsukami	Japan	3.2	4.6	3.9	7.7	7.8	7.8	150
Kawanakajima Hakuto	Japan	3.3	4.3	3.8	7.9	8.2	8.1	185
Chun Hua	Shanghai, China	3.0	4.3	3.7	6.3	6.7	6.5	115
Chun Mi	Henan, China	2.5	4.4	3.5	5.7	6.2	6.0	130
Springtime	USA	2.8	4.3	3.6	6.6	7.2	6.9	80
Da Guo Hei Tao	Shandong, China	2.7	3.8	3.3	6.5	6.5	6.5	146
Da Hong Pao	Hubei, China	2.6	4.1	3.4	6.4	6.8	6.6	108
Da Hong Tao	Shanxi, China	2.8	4.2	3.5	7.8	7.6	7.7	219
Okubo	Japan	2.7	4.1	3.4	7.5	7.1	7.3	159
Da Tian Tao	Jiangsu, China	2.6	4.2	3.4	6.7	7.6	7.2	158
Da Xue Tao	Hebei, China	3.2	5.2	4.2	9.4	10.0	9.7	126
Diao Zhi Bai	Anhui, China	3.0	4.5	3.8	7.1	7.8	7.5	130
Toobo	Japan	2.5	3.6	3.1	6.5	5.8	6.2	83
Azumo	Japan	3.4	4.8	4.1	7.0	6.9	7.0	100
Dunhuang Dong Tao	Gansu, China	3.0	3.9	3.5	7.1	6.2	6.7	104
Er Jie Bai	Henan, China	3.4	4.6	4.0	7.2	7.4	7.3	157
Er Zao Tao	Yunnan, China	2.3	3.9	3.1	6.2	6.1	6.2	93
Feicheng Bai Li 10	Shandong, China	3.0	4.8	3.9	7.2	7.2	7.2	191
Feicheng Bai Li 17	Shandong, China	3.3	4.9	4.1	8.2	8.3	8.3	258
Feicheng Hong Li 6	Shandong, China	3.2	4.9	4.1	9.4	8.4	8.9	221
Feng Bai	Liaoning, China	2.9	4.6	3.8	8.1	8.2	8.2	246
Feng Lu	Shanghai, China	2.8	4.6	3.7	7.8	7.3	7.6	115
Gan Xuan 4	Gansu, China	2.7	4.2	3.5	7.0	7.0	7.0	102
Okayama 3	Japan	2.5	3.8	3.2	6.7	7.4	7.1	130
Okayama 500	Japan	2.8	4.6	3.7	6.5	7.1	6.8	108
Hakuto	Japan	3.5	5.3	4.4	7.3	7.1	7.2	135
Okayama Wase	Japan	2.8	4.8	3.8	7.7	8.0	7.9	142
Gaotai 1	Gansu, China	3.1	4.6	3.9	7.4	7.7	7.6	133
Kouyou Hakuto	Japan	3.1	5.1	4.1	7.1	8.2	7.7	144
Ge Gu	Heibei, China	3.9	5.6	4.8	9.8	9.6	9.7	118
Guizhou Shui Mi	Guizhou, China	2.8	4.1	3.5	7.6	6.9	7.3	140
Han Lu Mi	Shandong, China	2.8	4.3	3.6	7.6	8.6	8.1	150
Hang Yu	Zhejiang, China	2.8	4.2	3.5	6.8	6.6	6.7	130
Hei Bu Dai	Henan, China	2.3	3.7	3.0	5.7	5.9	5.8	123
Hong Gan Lu	Liaoning, China	4.4	3.0	3.7	8.0	6.4	7.2	103
Hong Mei 2	Zhejiang, China	3.0	4.4	3.7	7.4	7.1	7.3	155
Hong Tao	Beijing, China	3.0	4.7	3.9	7.8	8.2	8.0	147
Hong Ya Zui	Heibei, China	2.6	3.9	3.3	6.6	7.2	6.9	104

Huichun Tao	Jilin, China	2.6	3.5	3.1	5.3	5.0	5.2	94
Huo Zhu	Jiangsu, China	2.3	3.6	3.0	5.9	6.5	6.2	117
Ji Zui Bai	Henan, China	3.0	4.6	3.8	8.0	8.5	8.3	145
Jilin 8501	Jilin, China	2.0	2.9	2.5	5.3	5.4	5.4	68
Jilin 8601	Jilin, China	2.1	2.9	2.5	4.8	5.6	5.2	50
Jilin 8701	Jilin, China	2.4	3.1	2.8	5.8	5.7	5.8	70
Jilin 8801	Jilin, China	1.9	2.7	2.3	4.8	5.1	5.0	46
Jian Zui Hong Rou	Henan, China	2.3	3.6	3.0	5.9	6.5	6.2	132
Jie Tu Bai	Heibei, China	3.0	4.4	3.7	8.4	8.4	8.4	220
Jingmen Tao	Hubei, China	2.5	3.5	3.0	6.7	6.5	6.6	108
Jiuyang Qing Tao	Guizhou, China	2.7	4.3	3.5	6.8	6.9	6.9	80
Kashi 4	Xinjiang, China	2.8	4.0	3.4	6.5	6.9	6.7	112
Laishan Mi	Shandong, China	2.8	4.4	3.6	7.2	8.0	7.6	193
Li He Tian Ren	Xinjiang, China	2.7	4.1	3.4	6.3	6.3	6.3	111
Lin Bai 3	Gansu, China	2.5	3.7	3.1	5.7	6.5	6.1	50
Lin Bai 10	Gansu, China	2.7	3.9	3.3	6.9	6.4	6.7	81
Liu Yue Bai	Heibei, China	3.0	4.3	3.7	7.5	8.0	7.8	114
Liu Yue Kong	Heinan, China	3.0	4.5	3.8	6.4	6.8	6.6	117
Robin	USA	2.6	3.9	3.3	6.9	6.8	6.9	92
Lulin Shui Mi	Jiangsu, China	2.7	4.0	3.4	7.2	7.5	7.4	131
Mi Yang Shan	Xinjiang, China	2.6	3.2	2.9	6.2	6.0	6.1	99
Nan Shan Tian Tao	Guangdong, China	2.3	3.6	3.0	6.3	6.6	6.5	100
Ping Bei Zi	Jiangsu, China	2.7	4.0	3.4	6.2	6.4	6.3	91
Pingyong Tao	Guizhou, China	2.1	2.8	2.5	5.4	5.4	5.4	73
Qi Zui Hong Rou	Henan, China	2.3	3.4	2.9	6.4	6.3	6.4	113
Qingling Dong Tao	Shaanxi, China	2.3	3.1	2.7	6.0	5.6	5.8	53
Qing Mao Zi Bai Hua	Sichuan, China	3.0	4.0	3.5	7.2	8.0	7.6	143
Qing Tao	Guizhou, China	2.8	4.7	3.8	7.7	8.6	8.2	160
Qingzhou Bai Pi Mi Tao	Shandong, China	1.9	3.0	2.5	5.2	5.9	5.6	46
Qingzhou Hong Pi Mi Tao	Shandong, China	2.1	2.9	2.5	5.8	5.2	5.5	52
Qiu Mi Shi Sheng	Shaanxi, China	2.9	4.2	3.6	8.2	7.5	7.9	222
Shenzhou Li He Shui Mi	Hebei, China	2.8	4.5	3.7	7.2	8.2	7.7	131
Shenzhou Shui Mi	Hebei, China	2.7	4.4	3.6	7.5	8.7	8.1	225
Shi Tou Tao	Henan, China	2.5	3.4	3.0	6.9	6.9	6.9	136
Shi Yu Bai Tao	Hebei, China	2.7	4.1	3.4	8.4	8.4	8.4	170
Tai Nong 2	Taiwan, China	2.1	3.1	2.6	6.5	6.0	6.3	78
Tianjin Shui Mi	Tianjin, China	2.6	4.1	3.4	7.2	8.5	7.9	100
Wanzhou Suan Tao	Chongqing, China	2.8	5.1	4.0	7.5	8.4	8.0	150
Wangmo Xiao Mi Tao	Guizhou, China	2.5	3.9	3.2	6.8	6.9	6.9	81
Wei Jian Hong Rou	Henan, China	2.4	4.0	3.2	5.8	6.6	6.2	127
Wu Da Tao	Guizhou, China	2.1	2.7	2.4	5.7	5.3	5.5	49
Wu Yue Xian	Beijing, China	2.7	4.3	3.5	7.5	8.0	7.8	112
Wuhan 2	Hebei, China	2.7	4.0	3.4	6.4	6.7	6.6	90
Xi Jiao 1	Shaanxi, China	3.1	4.3	3.7	7.2	7.7	7.5	135
Xi Mei 1	Shaanxi, China	2.5	4.3	3.4	6.3	6.5	6.4	112
Xi Mei 2	Shaanxi, China	2.7	4.2	3.5	7.6	7.1	7.4	100
Xi Nong 14-3	Shaanxi, China	2.7	4.5	3.6	7.0	7.8	7.4	150
Xi Nong 18	Shaanxi, China	2.9	4.6	3.8	8.5	8.1	8.3	154
Xi Nong 19	Shaanxi, China	2.6	3.6	3.1	7.0	6.4	6.7	100
Xiang Tao	Liaoning, China	2.6	3.3	3.0	7.0	7.0	7.0	136
Xingyi Bai Hua Tao	Guizhou, China	2.6	3.9	3.3	6.6	6.9	6.8	99
Yexian Dong Tao	Henan, China	2.2	2.9	2.6	5.6	5.7	5.7	55
Yi Xian Hong	Hebei, China	2.6	4.6	3.6	6.9	8.2	7.6	150
Ying Xue	Beijing, China	2.9	4.0	3.5	6.6	6.6	6.6	130
Yumyeong	Korea	3.0	4.5	3.8	7.3	7.2	7.3	190
Yu Hua Lu	Jiangsu, China	3.0	4.6	3.8	7.6	7.5	7.6	110
Yu Bai	Henan, China	2.7	4.4	3.6	6.7	7.3	7.0	130
Yu Tian	Henan, China	3.4	4.6	4.0	8.3	8.2	8.3	209
Yun Shu 2	Zhejiang, China	2.8	3.6	3.2	7.5	7.2	7.4	144
Zao Jiu Bao	Shanxi, China	3.0	4.7	3.9	7.2	7.3	7.3	115
Zao Xiang Yu	Beijing, China	2.3	3.8	3.1	6.0	6.2	6.1	123
Zao Yu	Beijing, China	2.1	3.0	2.6	6.5	7.7	7.1	260
Zhang Bai 2	Gansu, China	2.7	3.7	3.2	6.3	5.8	6.1	134
Zhang Bai 5	Gansu, China	2.8	4.5	3.7	7.7	7.2	7.5	125
Zheng Bai 5-2	Henan, China	2.5	3.7	3.1	6.7	7.5	7.1	112
Zheng Bai 5-38	Henan, China	3.0	4.0	3.5	7.1	6.6	6.9	250
Zhong Hua Shou Tao	Shandong, China	2.5	4.1	3.3	9.0	9.7	9.4	350
FAL16-33MR	USA	3.3	3.8	3.6	7.6	6.9	7.3	127
NJC77	USA	2.8	3.7	3.3	7.5	6.5	7.0	112
NJC112	USA	3.1	4.5	3.8	7.4	7.8	7.6	106
Elberta	USA	3.1	4.7	3.9	8.4	8.6	8.5	170
Cheng Xiang	Liaoning, China	2.3	3.3	2.8	6.8	7.1	7.0	95

Spring Gem	USA	2.8	3.8	3.3	7.1	7.3	7.2	105
Springgold	USA	2.0	2.8	2.4	4.9	5.2	5.1	74
Da Jin Dan	Yunnan, China	3.2	4.3	3.8	7.3	6.6	7.0	136
Da Li He Huang Rou	Xinjiang, China	3.0	3.9	3.5	6.5	6.2	6.4	108
Dalian 22-6	Liaoning, China	2.4	3.0	2.7	6.4	6.2	6.3	105
Dalian 22-8	Liaoning, China	3.1	4.4	3.8	8.1	8.2	8.2	181
Dawangzhuang Huang Tao	Henan, China	2.8	4.5	3.7	6.9	7.0	7.0	125
Da Zhao Huang Tao	Henan, China	3.0	4.2	3.6	7.7	7.3	7.5	121
Dicon	USA	2.6	4.2	3.4	7.7	8.1	7.9	162
Favolate 2	Italy	2.9	4.0	3.5	6.8	6.0	6.4	82
Favolate 3	Italy	2.7	4.0	3.4	6.8	6.9	6.9	88
Fay Elberta	USA	2.5	4.1	3.3	7.7	7.5	7.6	153
Phillips	USA	3.1	3.8	3.5	7.8	7.5	7.7	126
Feng Guan 2	Zhejiang, China	3.6	4.9	4.3	8.5	7.9	8.2	118
Fertilia Morettini	Italy	2.6	4.3	3.5	7.5	8.0	7.8	115
Fortuna	USA	1.1	3.7	2.4	6.8	6.2	6.5	91
Frederica	USA	2.7	3.7	3.2	7.3	6.0	6.7	130
Flordacrest	USA	2.7	3.6	3.2	7.4	6.9	7.2	120
Flordagold	USA	2.8	4.2	3.5	7.6	8.0	7.8	119
Gala	USA	3.0	4.5	3.8	7.5	7.7	7.6	138
Gan Xuan 2	Gansu, China	3.1	4.8	4.0	7.4	7.7	7.6	189
Gua Tao	Guangxi, China	2.1	3.6	2.9	6.5	6.4	6.5	101
Kanto 14	Japan	3.7	3.7	3.7	7.6	7.1	7.4	188
Blazeprince	USA	2.6	3.5	3.1	7.2	6.2	6.7	126
Flameprince	USA	2.9	4.6	3.8	8.4	8.4	8.4	206
Guizhou Huang Jin Mi	Guizhou, China	2.6	3.6	3.1	7.5	7.3	7.4	143
Harbrite	Canada	2.4	4.1	3.3	6.8	7.6	7.2	120
Harken	Canada	2.3	3.4	2.9	7.6	7.1	7.4	160
Havis	USA	3.0	4.3	3.7	9.1	8.5	8.8	211
Hanthorne	USA	3.4	4.9	4.2	8.6	7.6	8.1	212
Hetian Huang Rou	Xinjiang, China	2.5	3.1	2.8	6.2	5.6	5.9	70
Harmony	USA	3.3	5.3	4.3	8.3	8.1	8.2	155
Redtop	USA	2.4	3.9	3.2	7.6	7.4	7.5	133
Redskin	USA	2.6	3.8	3.2	7.9	6.9	7.4	143
Huang La Tao	Guangxi, China	2.9	4.0	3.5	6.4	6.9	6.7	142
Huang Yan	Yunnan, China	2.9	4.3	3.6	7.0	7.1	7.1	123
Jiangcun 4	Gansu, China	3.0	3.6	3.3	7.3	6.7	7.0	151
Jiangcun 5	Gansu, China	3.2	4.2	3.7	8.0	9.0	8.5	159
Jin Lu	Liaoning, China	2.9	4.8	3.9	7.2	6.8	7.0	144
Marigold	USA	2.6	3.7	3.2	6.5	6.0	6.3	88
Jin Shi Ji	Hebei, China	2.8	3.9	3.4	7.7	7.9	7.8	300
Babygold 6	USA	2.8	4.0	3.4	8.0	7.2	7.6	144
Babaygold 7	USA	2.8	4.2	3.5	8.0	7.8	7.9	156
Dwarf Norman	USA	2.4	4.5	3.5	7.7	8.2	8.0	193
Cullinan	USA	2.7	4.2	3.5	7.7	6.9	7.3	200
Lafeliciana	USA	2.6	3.7	3.2	6.5	6.0	6.3	106
Lian Huang	Liaoning, China	2.5	4.7	3.6	7.7	7.7	7.7	165
Long 1-2-6	Gansu, China	2.9	4.8	3.9	6.8	7.5	7.2	136
Long 2-4-6	Gansu, China	2.9	3.7	3.3	6.5	6.7	6.6	128
Norman	USA	2.7	4.3	3.5	7.7	7.3	7.5	139
Rumiana	保加利亚	3.5	4.3	3.9	8.5	8.2	8.4	180
McNeely	USA	2.7	4.4	3.6	8.0	7.8	7.9	200
Mei Jin	Heibei, China	2.0	3.5	2.8	7.0	7.2	7.1	150
Myojo	Japan	3.0	4.3	3.7	7.6	7.8	7.7	166
Moyu 8	Xinjiang, China	2.4	3.5	3.0	5.7	5.8	5.8	91
Rio Oso Gem	USA	2.5	4.3	3.4	7.5	7.5	7.5	114
Regina	USA	2.7	4.3	3.5	6.7	7.0	6.9	130
Qing Si	Yunnan, China	2.7	4.1	3.4	7.0	6.2	6.6	126
Autumn Prince	USA	2.8	4.0	3.4	7.8	7.3	7.6	142
Tropicbeauty	USA	2.4	3.3	2.9	6.4	5.7	6.1	95
Texroyal	USA	2.7	3.4	3.1	7.5	6.7	7.1	108
Texstar	USA	3.0	3.9	3.5	7.6	6.9	7.3	104
Tugou 1	Gansu, China	2.3	3.3	2.8	5.5	5.4	5.5	92
Wan Huang Jin	Japan	3.3	4.6	4.0	8.3	8.9	8.6	186
Flavorcrest	USA	2.1	3.2	2.7	6.6	6.5	6.6	142
Gold	Japan	2.6	4.1	3.4	7.9	8.5	8.2	208
Xiang Jiao Tao	Liaoning, China	2.9	4.1	3.5	6.9	7.2	7.1	117
Zao Huang Huan	Shaanxi, China	2.8	4.8	3.8	7.2	7.8	7.5	122
Improved Flavor Crest	USA	2.5	3.5	3.0	6.1	6.1	6.1	150
Zheng Huang 15-1	Henan, China	2.8	4.5	3.7	7.2	7.2	7.2	132
Zheng Huang 4	Henan, China	3.4	5.2	4.3	9.4	9.0	9.2	222
NJN69	USA	2.9	4.1	3.5	6.9	6.6	6.8	115

NJN70	USA	2.5	4.1	3.3	5.8	5.9	5.9	108
NJN76	USA	3.1	4.0	3.6	7.1	6.9	7.0	130
NJN89	USA	2.6	4.1	3.4	6.8	6.0	6.4	108
NJN93	USA	3.1	4.6	3.9	8.3	7.8	8.1	112
Maria Emilia	Italy	2.5	4.3	3.4	6.1	6.5	6.3	93
Anderson	Italy	2.6	4.2	3.4	6.8	6.6	6.7	95
Bulgaria 2	保加利亚	2.7	4.3	3.5	6.6	6.4	6.5	110
Arctic Queen	USA	2.7	3.5	3.1	6.8	6.7	6.8	157
Arctic Rose	USA	2.5	3.7	3.1	6.9	6.3	6.6	130
Arctic Star	USA	2.6	3.4	3.0	6.6	6.2	6.4	137
Troubadour	France	2.7	3.7	3.2	6.9	6.3	6.6	120
Super Crimson	USA	2.3	3.3	2.8	5.9	5.4	5.7	76
Dalian 60-14-111	Liaoning, China	3.0	4.7	3.9	7.0	7.0	7.0	95
Croce Decsus	USA	3.0	5.0	4.0	6.6	7.1	6.9	83
Fairlane	USA	3.1	4.6	3.9	7.1	6.8	7.0	139
Fuzador	France	2.4	3.5	3.0	6.5	6.2	6.4	94
Fuzalode	France	2.6	3.3	3.0	6.9	6.0	6.5	88
Nectagrand 2	Italy	2.3	3.8	3.1	6.4	6.1	6.3	85
Nectagrand 7	Italy	2.8	4.4	3.6	7.3	7.4	7.4	113
Sunraycer	USA	2.4	3.3	2.9	6.7	6.4	6.6	85
Harko	USA	2.4	3.6	3.0	5.8	5.1	5.5	80
Hardired	USA	3.0	4.5	3.8	8.4	8.2	8.3	128
Heyang You Tao	Shaanxi, China	2.9	3.9	3.4	7.0	6.6	6.8	104
Red Diamond	USA	2.6	3.8	3.2	6.3	5.9	6.1	94
Redgold	USA	2.6	3.8	3.2	6.8	7.0	6.9	148
Sunred	USA	2.0	2.8	2.4	5.3	5.3	5.3	60
Hong Shan Hu	Beijing, China	2.8	4.0	3.4	7.1	6.5	6.8	151
Nactared 4	USA	2.4	3.7	3.1	6.0	5.7	5.9	79
NJN26	USA	2.6	4.1	3.4	6.7	6.4	6.6	85
Hu You 004	Shanghai, China	2.6	3.9	3.3	6.5	6.9	6.7	127
Imoiouniku	Japan	2.4	3.4	2.9	5.8	5.7	5.8	96
September Free	USA	2.6	3.7	3.2	6.3	5.8	6.1	107
Kashi Huang Rou Li Guang	Xinjiang, China	2.8	3.6	3.2	7.0	6.1	6.6	86
Maria Carla	Italy	2.9	4.7	3.8	6.5	7.0	6.8	108
Le Yuan	Henan, China	2.9	4.0	3.5	7.1	6.9	7.0	116
Fantasia	USA	2.8	4.0	3.4	7.2	6.8	7.0	150
Legrand	USA	2.8	4.4	3.6	6.6	6.8	6.7	151
June Prince	USA	2.8	4.2	3.5	6.1	6.3	6.2	101
Roseprincess	USA	2.9	4.1	3.5	7.2	6.7	7.0	127
Mei Gui Hong	Henan, China	2.6	3.8	3.2	6.9	6.0	6.5	123
Flavortop	USA	3.0	4.3	3.7	7.5	7.2	7.4	150
Summer Beauty	USA	3.0	4.0	3.5	7.0	6.4	6.7	92
Croce Del Sud	Italy	2.9	4.3	3.6	6.6	6.9	6.8	72
Hiratsuka Red	Japan	2.7	4.0	3.4	6.6	6.8	6.7	100
Qin Guang	Shaanxi, China	2.8	4.3	3.6	6.6	7.3	7.0	108
Qin Guang 2	Shaanxi, China	2.6	4.0	3.3	7.3	7.2	7.3	147
Sungold	USA	2.9	4.4	3.7	7.5	6.7	7.1	140
Rui Guang 3	Beijing, China	3.1	4.3	3.7	7.3	7.2	7.3	150
Rui Guang 7	Beijing, China	2.5	3.4	3.0	5.7	5.7	5.7	110
Rui Guang 19	Beijing, China	2.8	3.6	3.2	6.7	6.1	6.4	110
SunGlo	USA	3.0	4.4	3.7	7.8	7.1	7.5	137
Sunfre	USA	2.9	3.8	3.4	6.5	6.1	6.3	117
Shuang Xi Hong	USA	2.7	3.8	3.3	6.8	6.6	6.7	140
Suan Li Guang	Xinjiang, China	2.8	3.6	3.2	6.7	6.0	6.4	122
Cheonhong	Korea	2.5	3.9	3.2	7.3	7.1	7.2	123
Tian Li Guang	Xinjiang, China	2.7	3.2	3.0	5.8	6.2	6.0	95
Venus	Italy	3.4	4.6	4.0	8.2	7.2	7.7	150
Winberger	Italy	2.7	4.0	3.4	5.9	5.9	5.9	82
May Grand	USA	3.0	4.3	3.7	8.0	7.2	7.6	142
Summer Grand	USA	3.0	4.3	3.7	6.4	6.4	6.4	110
Okitsu	Japan	2.8	4.3	3.6	7.3	8.4	7.9	114
Snow Queen	USA	2.8	4.1	3.5	6.5	6.5	6.5	125
Sunsplash	USA	2.7	3.7	3.2	6.5	6.1	6.3	78
Early Red 2	USA	3.0	3.8	3.4	7.0	6.3	6.7	121
Early Grand	USA	2.7	4.2	3.5	6.4	6.8	6.6	79
Zheng You Zi Hong Tao	Henan, China	2.9	3.9	3.4	6.1	5.9	6.0	77
Zhong Nong Jin Hui	Henan, China	2.8	4.2	3.5	6.9	6.8	6.9	173
Zhong You Tao 8	Henan, China	3.0	4.4	3.7	7.8	7.4	7.6	174
Bai Dan Ban	East China	2.1	3.6	2.9	6.2	5.7	6.0	?
Bao Chun	Henan, China	2.2	3.0	2.6	4.7	4.6	4.7	?
Beijing S9	Beijing, China	2.9	3.8	3.4	6.8	7.8	7.3	150
Dan Ban Zi Tao	North China	2.1	3.5	2.8	5.9	6.2	6.1	?

Fen Shou Xing	Jiangsu, China	2.4	3.2	2.8	5.8	5.8	5.8	90
Hong Chui Zhi	North China	1.9	3.1	2.5	3.9	4.0	4.0	?
Hong Hua Bi Tao	North China	1.8	2.8	2.3	4.0	4.3	4.2	?
Hong Ju Hua Tao	Henan, China	1.8	3.1	2.5	4.0	4.9	4.5	?
Hong Shou Xing	North China	2.0	3.0	2.5	3.6	3.7	3.7	?
Hong Ye Tao	North China	2.6	3.7	3.2	5.9	6.2	6.1	?
Hua Yu Lu	Zhejiang, China	2.6	3.5	3.1	6.9	7.1	7.0	116
Huang Jin Mei Li	USA	3.0	4.5	3.8	8.5	9.5	9.0	171
Jiang Tao	USA	2.1	3.1	2.6	5.7	6.0	5.9	?
Ju Hua Tao	North China	1.8	3.1	2.5	3.9	4.7	4.3	
Man Tian Hong	Henan, China	3.1	4.0	3.6	7.5	8.3	7.9	148
Ren Mian Tao	North China	2.6	4.2	3.4	5.4	6.1	5.8	?
Shou Bai	Beijing, China	1.8	2.9	2.4	4.4	4.4	4.4	?
Shou Fen	Beijing, China	1.8	3.0	2.4	4.7	4.9	4.8	?
Zhu Fen Chui Zhi	North China	1.8	2.7	2.3	3.4	4.0	3.7	?
Okinawa	USA	2.0	2.8	2.4	4.3	4.3	4.3	36
Bailey	USA	2.5	3.4	3.0	6.7	6.4	6.6	79
Harrow Blood	Canada	1.8	2.8	2.3	5.2	5.1	5.2	47
Ha Lu Hong Shi Sheng	Henan, China	2.7	3.4	3.1	7.3	6.6	7.0	106
Rutgers Redleaf	USA	2.3	3.3	2.8	5.5	5.3	5.4	60
Siberian C	Canada	2.1	3.1	2.6	4.1	4.4	4.3	28
Tsukuba 2	Japan	2.0	3.0	2.5	5.1	4.6	4.9	53
Tsukuba 3	Japan	2.4	3.8	3.1	6.1	6.9	6.5	88

Result of Radiocarbon Dating Analysis

Sample Data	Measured Radiocarbon Age	^{13}C / ^{12}C Ratio	Conventional Radiocarbon Age
Beta - 354489	NA	-27.5 o/oo	> 43500 BP

SAMPL : Ciying001
ANALYSIS : AMS-Standard delivery
MATERIAL/PRETREATMENT : (nutshell): acid/alkali/acid
COMMENT:

(1) A Measured Radiocarbon Age is not reported for infinite dates since corrections may imply a greater level of confidence than is appropriate.

(2) The ^{14}C activity was extremely low and almost identical to the background signal. In such cases, indeterminate errors associated with the background add non-measurable uncertainty to the result. Always, the result should be considered along with other lines of evidence. The most conservative interpretation of age is infinite (i.e. greater than).

Result of Elemental Analysis

Element	Line Type	Apparent Concentration	k Ratio	Wt%	Wt% Sigma	Atomic %	Standard Label	Factory Standard
C	K series	0.32	0.00321	3.21	0.20	7.53	C Vit	Yes
O	K series	13.02	0.04381	33.52	0.77	58.94	SiO2	Yes
Mg	K series	0.07	0.00045	0.41	0.13	0.47	MgO	Yes
Al	K series	0.14	0.00099	0.86	0.16	0.89	Al2O3	Yes
Si	K series	0.21	0.00169	1.38	0.19	1.38	SiO2	Yes
Ca	K series	0.18	0.00157	1.29	1.56	0.91	Wollastonite	Yes
Fe	L series	5.69	0.05687	59.32	1.15	29.88	Fe	Yes
Total:				100.00		100.00		
